# Who gains the most from improving working conditions? Health-related absenteeism and presenteeism due to stress at work

**DOI:** 10.1007/s10198-019-01084-9

**Published:** 2019-07-15

**Authors:** Beatrice Brunner, Ivana Igic, Anita C. Keller, Simon Wieser

**Affiliations:** 1 Winterthur Institute of Health Economics, Zurich University of Applied Sciences, Gertrudstrasse 15, 8401 Winterthur, Switzerland; 2 Department of Work and Organizational Psychology, University of Bern, Fabrikstrasse 8, 3012 Bern, Switzerland; 3 Department of Organizational Psychology, University of Groningen, Grote Kruisstraat 2/1, 9712 TS, Groningen, The Netherlands

**Keywords:** Health-related productivity losses, Task-related and social stressors and resources at work, Self-efficacy, Absenteeism, Presenteeism, J22, J24, I10, I15

## Abstract

Work stress-related productivity losses represent a substantial economic burden. In this study, we estimate the effects of social and task-related stressors and resources at work on health-related productivity losses caused by absenteeism and presenteeism. We also explore the interaction effects between job stressors, job resources and personal resources and estimate the costs of work stress. Work stress is defined as exposure to an unfavorable combination of high job stressors and low job resources. The study is based on a repeated survey assessing work productivity and workplace characteristics among Swiss employees. We use a representative cross-sectional data set and a longitudinal data set and apply both OLS and fixed effects models. We find that an increase in task-related and social job stressors increases health-related productivity losses, whereas an increase in social job resources and personal resources (measured by occupational self-efficacy) reduces these losses. Moreover, we find that job stressors have a stronger effect on health-related productivity losses for employees lacking personal and job resources, and that employees with high levels of job stressors and low personal resources will profit the most from an increase in job resources. Productivity losses due to absenteeism and presenteeism attributable to work stress are estimated at 195 Swiss francs per person and month. Our study has implications for interventions aiming to reduce health absenteeism and presenteeism.

**Electronic supplementary material:**

The online version of this article (10.1007/s10198-019-01084-9) contains supplementary material, which is available to authorized users.

## Introduction

A loss of work productivity can be a result of health impairments and arise from absenteeism (being away from work due to illness or disability) and presenteeism (being present at work but constrained in certain aspects of job performance by health problems) [1]. Maintaining a healthy and productive workforce is increasingly challenging due to the continuing structural changes in the working environment, an aging workforce and an increasing number of employees affected by stress at work [2]. Gaining better knowledge of the stress-related causes of absenteeism and presenteeism is therefore of high social and economic importance. A detailed analysis of the drivers of work stress-related productivity losses may be particularly useful to understand which employees are most at risk of incurring stress-related productivity losses and to identify those who might profit the most from interventions that improve work conditions.

Productivity losses are determined by multiple factors [3], but work-related factors have often been proposed as especially important [4]. According to the models developed in occupational health psychology, such as the Job-Demands Control model (JDC) [5, 6] and the Job-Demands Resources model (JDR) [7], unfavorable job conditions are associated with high levels of job stressors and a lack of job resources. Exposure to such job conditions can lead to stress among employees, resulting in decreased performance and motivation and, over time, in serious health problems [8]. However, only a handful of empirical studies have analyzed these propositions in relation to productivity losses caused by absenteeism and presenteeism (e.g., [4, 9, 10]). For example, a lack of job control, which is a well-established work resource [11] defined as the ability to determine when and where work is done, has been shown to increase the risk of presenteeism [12]. Another study found a similar relation with sickness absence but only for women [13]. Additionally, high time demands and physical demands at work have been shown to be associated with presenteeism and absenteeism [14, 15].

In this study, we estimate the effects of work stressors and resources on health-related productivity losses caused by absenteeism and presenteeism, and add to the current literature in three ways.

*First*, we estimate the effects of task-related and social job stressors and resources on health-related productivity losses, whereas the current literature mainly focused on task-related factors. Empirical evidence suggests that social stressors may be especially harmful to employee health and well-being, even more so than other job stressors [16]. Furthermore, the recent “Stress-as-Offense-to-Self” model underlines the relevance of social job resources, such as appreciation at work, and highlights its absence as particularly stressful for employees [17]. Absenteeism and presenteeism can also be explained by the social exchange perspective [18]. According to this approach, the employee–organization relationship is a trade of effort and loyalty for benefits such as pay, social support and recognition [19]. When employees are satisfied with this mutual exchange, they will be engaged in their jobs. However, when employees perceive the benefits received as too low compared to their contribution, they may withdraw from the relationship. Absenteeism and presenteeism can thus be seen as a method of restoring equity in the employee–organization relationship [20]. Some previous studies support these assumptions, although evidence is still scarce. Injustice at work [20], low organizational support [18] and low workgroup cohesiveness [21] have, for example, been shown to increase the risk of absenteeism. Similarly, negative relationships with colleagues [22], role ambiguity [23], and workplace bullying [24] have been shown to increase the risk of presenteeism. A few studies also provide evidence of the relevance of positive social aspects at work. Employees working under a supportive supervisor [25] who demonstrated strong integrity [26] showed less presenteeism and absenteeism.

*Second*, in addition to social and task-related stressors and resources at work, we consider personal resources. According to the JDR model, job and personal resources can affect health and organizational outcomes both directly and indirectly; personal resources might enable employees not only to deal with job demands in a resilient way but also to make better use of available job resources [7]. Previous studies have shown that personal resources are related to absenteeism; however, studies on presenteeism rarely consider personal factors [27]. We included occupational self-efficacy as a relevant personal resource for individuals in organizations [28, 29], expecting self-efficacy to act as a buffer for the negative effects of job stressors [7]. Occupational self-efficacy is defined as the belief or confidence in one’s ability to successfully fulfill a task or cope with difficult tasks or problems [30]. Previous research has shown direct beneficial effects of occupational self-efficacy on productivity [28, 29], work-related behavior [31] and job attitudes [32] and demonstrated its moderating effects in the stressors–strain relationship [33, 34]. However, to date, no studies have explored its effects on health-related productivity losses. Based on the conservation of resources theory (COR, [35]), according to which individuals who lack resources are more vulnerable to resource loss and less capable of resource gain (negative spiral), we expected that employees lacking both job and personal resources are most at risk of experiencing health-related productivity losses due to absenteeism and presenteeism when job stressors increase. Furthermore, in line with the “gain paradox principle” [35] according to which resource gains become more important when resources are loss is high, we expect that an increase in job resources is especially important for employees with low self-efficacy and high stressors.

*Third*, we contribute to the literature on the economic burden of work stress. Although work stress and its consequences for employees and employers are high on the political agendas of European institutions and policy-makers [36], evidence of the economic burden of work stress is scant, especially regarding stress-related presenteeism [37]. The few available studies suggest that the costs of work stress are substantial [38]. We add to previous studies on the productivity losses caused by work stress by estimating the cost of employees’ health-related productivity loss due to presenteeism and absenteeism of being exposed to an imbalance between job stressors and job resources. Such an imbalance, according to occupational stress models (e.g., JDC, JDCR [39]), results in work stress and has a high probability of leading to serious health problems. We calculated the total health-related productivity loss due working under unfavorable job conditions per employee and month, considering both absenteeism and presenteeism.

The aim of this study was threefold. *First*, we estimated the effects of task-related and social stressors and resources on health-related productivity losses due to absenteeism and presenteeism. We assessed stressors and resources at work based on six indices measuring (1) task-related work stressors (time pressure, task uncertainty, performance constraints, and mental and qualitative overload), (2) social work stressors (social stressors from supervisor and co-workers), (3) task-related work resources (job control and task significance), (4) social work resources (social support from supervisor, appreciation at work), as well as (5) overall work stressors and (6) overall work resources. We controlled for a wide range of confounding factors, such as socio-economic characteristics, job characteristics, private demands, and personal characteristics (self-efficacy). *Second*, we explored the interaction effects between job stressors, job resources, and personal resources. We aimed to understand which employees are most at risk if job stress increases and which employees would benefit the most from interventions improving the balance between job stressors and resources. *Third*, we built an economic model estimating the productivity losses caused by employee exposure to an imbalance between job stressors and resources.

## Methods

### Data

We used data from a Swiss workforce survey carried out in two measurement waves. The survey consisted of two datasets: a representative cross-sectional dataset based on the first wave and a longitudinal dataset based on both waves. We used both datasets because they have different strengths and weaknesses. The cross-sectional wave 1 dataset is representative of the Swiss workforce regarding gender, age, region and industry branch. Moreover, it contains information on occupational self-efficacy, allowing us to explore the interaction effects between job stressors, job resources and occupational self-efficacy. However, due to its cross-sectional nature, it could not be used to identify causal effects. The longitudinal wave 1–2 dataset allowed us to overcome this weakness, as it permits the application of methodologically superior panel data estimation methods. However, wave 1–2 suffers from considerable attrition in the second wave and does not allow us to explore interaction effects because occupational self-efficacy was not assessed in the second wave.

*Wave 1* The first wave was conducted in February 2014. The recruitment of participants was based on a large Swiss Internet panel including full- and part-time employees. The sample was stratified by gender, age, region and industry branch. Participants were recruited randomly from the sample by phone and e-mail to complete the online questionnaire [40]. A total of 3758 employees completed the questionnaire. Of these, 59 were excluded because of timing and response patterns and 318 because of missing or implausible information. The final cross-sectional sample consisted of 3381 employees who are representative of the Swiss workforce regarding gender, age, region and industry branch.

*Wave 1–2* The second wave was conducted in February 2015. Of the 3381 participants of wave 1, 352 had left the panel and 196 were no longer economically active and were therefore excluded. Hence, 2833 individuals were re-contacted, of whom 2125 (75%) participated and 1759 (62%) completed the questionnaire. We excluded 93 individuals because of timing and response patterns and 153 because of missing information on industry branch or work productivity. Plausibility checks on income and hours worked led to the exclusion of an additional 14 individuals. Our final longitudinal sample included *N* = 1513 individuals who had participated in both waves. The longitudinal wave 1–2 data set was used to test the robustness of the cross-sectional estimations. We accounted for selective attrition by estimating and applying inverse-probability-of-attrition weights.

### Measures

#### Dependent variable

Our dependent variable was individual *health*-*related productivity loss*, corresponding to the sum of the percentage of absenteeism (percentage of work time missed due to health) and percentage of presenteeism (percentage of work time affected by productivity impairment due to health problems while working). These data were collected with the Work Productivity and Activity Impairment-General Health (WPAI-GH) questionnaire. The WPAI-GH is a psychometrically tested instrument measuring absenteeism, presenteeism and overall health-related work productivity losses (corresponding to the sum of absenteeism and presenteeism) with good reliability, validity, generalizability and practicability [41–43].

The WPAI-GH questionnaire is composed of five questions: Q1 = currently employed; Q2 = hours missed due to health problems; Q3 = hours missed due to other reasons (e.g., vacation); Q4 = hours actually worked; Q5 = degree to which health affected productivity while working (using a 0–10 Visual Analogue Scale). Following the coding and scoring rules of the WPAI developers, we obtained the percentage of health-related work productivity losses [Q2/(Q2 + Q4) + ((1 − Q2/(Q2 + Q4)) × Q5/10)], which constitutes our dependent variable.

The main advantages of the WPAI over other productivity questionnaires [e.g., health and work questionnaire (HWQ) or health and work performance questionnaire (HPQ)] are the possibility of transforming outcomes into monetary values, as outcomes are expressed as impairment percentages. Furthermore, it uses a 1-week rather than a 4-week recall period, which significantly reduces recall bias [42].

#### Main explanatory variables

Our main explanatory variables are six indices measuring task-related and social stressors and resources at work. The indices were constructed based on several task-related and social work conditions proposed by the theoretical and empirical literature to be relevant regarding health and key organizational variables such as productivity and motivation (e.g., JDC, JDCR [7, 8, 11]). We included four well-established task-related stressors (time pressure, task uncertainty, performance constraints, and mental and qualitative overload) and two resources (job control and task significance). In addition to the task-related factors, we included two social resources (social support from supervisor, appreciation at work) [25, 44, 45] and two social stressors (social stressors from supervisor and co-workers) [46, 47] as suggested by theory [8] and empirical research [48, 49]. We also included occupational self-efficacy, a personal resource, to test proposed interaction effects [33, 34].

##### Job stressors

We assessed four *task*-*related stressors* with items from the Instrument for Stress-Oriented Task Analysis (ISTA; [50]), including *time pressure* (e.g., “How often must you finish work later because of having too much to do?”), *task uncertainty* (e.g., “How often do you receive contradictory instructions from different supervisors?”), *performance constraints* (e.g., having to work with inadequate devices or obsolete information) [50], and *mental and qualitative overload* at work (three items, e.g., having to perform tasks that exceed one’s skills) [51]. With the exception of the last scale, which has three items, each of the scales contains four items. We assessed *social stressors* with the social stressors scale by Frese and Zapf, which includes two scales each with five items. One scale focuses on *conflicts or animosities and negative group climate among co*-*workers* (e.g., “With some colleagues there is often conflict”), and the other focuses on *conflicts with supervisors* (e.g., “I often quarrel with my boss”) [52].

##### Job resources

We assessed *job control* using the ISTA [50]. The five items measured job control by evaluating respondents’ freedom to choose the time (e.g., “To what degree are you able to decide on the amount of time you will be working on a certain task?”) and method (e.g., “Can you decide yourself which way to carry out your work?”) for accomplishing tasks at work. The second task-related resource was *task significance* (“In my job, one can produce something or carry out an assignment from A to Z”), which was measured with one item from the *Salutogenetische Subjektive Arbeitsanalyse* (*SALSA;* [51]) instrument. Social job resources were measured by four items evaluating *supportive behavior from supervisors* (e.g., a line manager lets a worker know how well a job was done), which was also measured with items from the *SALSA* [51], and *appreciation at work*, which was assessed with a single item based on the *Appreciation at Work Scale* (“I feel generally appreciated in my job”) [53]. With the exception of appreciation, all items were answered on a 5-point Likert scale, with responses ranging from 1 (very little/not at all) to 5 (very much). Appreciation, originally answered on a 7-point Likert scale, was transformed into a 5-point scale.

##### Personal resources

We assessed *occupational self*-*efficacy* with a four-item scale from Rigotti, Schyns, and Mohr [54]*. Work*-*related self*-*efficacy* measures the belief in one’s ability to cope with difficult tasks and problems at work (e.g., “I can remain calm when facing difficulties in my job because I can rely on my abilities”) and was assessed only in the first wave of the survey (wave 1).

We created six indices measuring the level of job demands and job resources. *First*, to test the overall effects of job stressors and resources on employees’ health-related productivity losses, we built an overall job stressors and an overall job resources measure. These measures were constructed by averaging over all six stressors and four resources described above, representing demands and resources from the JDC model. This procedure has been previously used [55]. *Second*, to test the distinct productivity effects of task-related and social stressors and resources, we constructed four additional indices measuring (1) task-related stressors, (2) social stressors, (3) task-related resources and (4) social resources. The four measures were constructed similarly, by averaging over the single task-related and social job stressors and resources. Table A.1 presents the Cronbach alpha values for the single stressors and resources as well as for the indices. For the analysis, we used the standardized values of the six job stressor and resource measures.

### Covariates

We considered a variety of potential confounders that, based on previous evidence, were expected to be associated with work productivity as well as job stressors and resources. *First*, we controlled for several demographic and socio-economic characteristics (gender, age, number of children, marital status, educational level, and whether the respondent had Swiss citizenship) [56, 57]. *Second*, we controlled for labor market and job characteristics such as industry branch, occupation, company size, job tenure, average number of working hours, shiftwork, part-time employment, and managerial function, as has been done in previous studies [56, 58, 59]. *Third*, we controlled for chronic physical health conditions such as asthma, allergies, cancer, chronic bronchitis or emphysema, diabetes, kidney disease, osteoarthritis or rheumatoid arthritis, osteoporosis, and permanent injury after an accident, as the negative relationship between health problems and work productivity is well established [60, 61]. We did not, however, consider diseases with often psychosomatic causes, such as migraines or depression, as they may be a part of the outcome [11, 62] and therefore represent bad control variables for our research question [63]. *Finally*, we controlled for family-to-work conflict [64] to account for the potential productivity effects of mood spillovers, which have been identified in previous studies [65].

## Econometric framework

Since our analysis was based on a cross-sectional dataset (wave 1) and on a longitudinal dataset (wave 1–2), we applied both cross-sectional and panel-data estimation methods. Cross-sectional methods were used to explore the association of health-related productivity loss with job stressors and job resources as well as to explore the interaction between job stressors, job resources, and personal resources in wave 1. The wave 1 dataset is representative of the Swiss workforce and holds information on occupational self-efficacy, which the second wave does not. However, cross-sectional estimation methods require the key regressors to be strictly exogenous conditional on covariates in order to have a causal interpretation. Panel data methods allow for relaxing this strong assumption by controlling for unobserved time-invariant heterogeneity. We used the wave 1–2 panel data set to test the robustness of the cross-sectional estimation results estimating fixed effects models while accounting for selective attrition using inverse-probability-of-attrition weights.

### Cross-sectional estimation

The associations between health-related productivity losses and job stressors and resources were examined based on five model specifications with hierarchical adjustment and estimated by ordinary least squared (OLS). The fully specified model takes the following form:

1 $$\begin{aligned} Y_{i} & = \alpha + \beta_{1} \dot{R}_{i}^{j} + \beta_{2} \dot{S}_{i}^{j} + \gamma {\mathbf{X}}'_{i} + \delta {\mathbf{J}}'_{i}\\ & \quad + \varphi_{\text{r}} + \mu \dot{S}_{i}^{p} + \theta {\mathbf{H}}'_{i} + \vartheta \dot{R}_{i}^{p} + \varepsilon_{i} , \\ & \quad {\text{with}}\quad \dot{R}_{i}^{j} \in \left( {\dot{R}_{i}^{j} ,(\dot{R}_{i}^{{j,{\text{social}}}} ,\dot{R}_{i}^{{j,{\text{task}}}} ) } \right)\\ & \quad\quad {\text{and}}\quad \dot{S}_{i}^{j} \in \left( {\dot{S}_{i}^{j} , (\dot{S}_{i}^{{j,{\text{social}}}} ,\dot{S}_{i}^{{j,{\text{task}}}} )} \right). \\ \end{aligned} ,$$

$Y_{i}$ denotes the percentage productivity losses of individual *i* due to sickness absenteeism and presenteeism. $\dot{R}_{i}^{j}$ and $\dot{S}_{i}^{j}$ represent the level of resources and stressors at individual *i*’s current job (the dots representing standardized values). The fully specified model distinguishes between task-related and social job stressors and resources. The hierarchical adjustment involves the following five model specifications. We started by estimating simple correlations with Model 1 (CS-1), including only job resources $(\dot{R}_{i}^{j} )$ and job stressors $(\dot{S}_{i}^{j} )$. Model 2 (CS-2) additionally included known confounding variables related to socio-economic and job characteristics (${\mathbf{X^{\prime}}}$ and ${\mathbf{J^{\prime}}}$, see *covariates* section for more details). This model also included regional fixed effects, denoted by $\varphi_{\text{r}}$ with r indexing the canton of residence of individual *i*. Model 3 (CS-4) additionally included family-related stressors ($\dot{S}_{i}^{p}$), as previous literature suggests that mood disturbances can spill over from the family domain to the work domain [65]. Model 4 (CS-4) added a set of nine dummy variables indicating chronic health conditions (${\mathbf{H^{\prime}}}$, see “Covariates” section) to account for the relationship between chronic conditions, such as asthma and diabetes, and work productivity [60, 61]. Finally, our fully specified Model 5 (CS-5) included occupational self-efficacy ($\dot{R}_{i}^{p}$). Under the assumption of strict exogeneity, $\beta_{1}$ and $\beta_{2}$ represent the percentage-point change in health-related productivity losses due to a one-standard-deviation change in job resources and job stressors. Note that there is a potential issue of reverse causality. However, this problem is likely to be mitigated as the dependent variable referred to the week before the interview, while the key regressors referred to the current work situation in general and may therefore be considered predetermined.

### Panel data estimation and inverse-probability-of-attrition weighting

We assessed the robustness of the fully specified cross-sectional model (CS-5) by estimating a fixed effects model based on the longitudinal wave 1–2 dataset while accounting for selective attrition using inverse-probability-of-attrition weighting. The fixed effects model differed from the fully specified cross-sectional model in Eq. (1) in three ways. *First*, it included individual fixed effects. This allowed us to relax the assumption of strict exogeneity as the model controlled for unobserved time-invariant heterogeneity. *Second*, it excluded occupational self-efficacy because it was not observed in the second wave. While the time-invariant component of occupational self-efficacy was captured by the individual fixed effect, we could not control for its time-variant component, i.e., potential productivity effects resulting from changes in occupational self-efficacy. However, self-efficacy is considered to be stable over time [66]. *Third*, for the fixed effects model to provide unbiased estimates of $\beta_{1}$ and $\beta_{2}$, it is essential to avoid attrition bias; thus, the fixed effects model weights observations by inverse-probability-of-attrition weights.

Inverse-probability-of-attrition weighting involved two steps. *First*, for each period with potential selective attrition (in our case, wave 2), a dummy variable indicating second-wave participation was regressed on a series of covariates in wave 1, and probabilities $\hat{P}_{i2}$ were fitted using logistic regression. The covariates also included variables on attitudes, character traits, mental health and well-being, and many other variables not used in Eq. (1) (see Appendix A.2 for specification details). In the *second* step, the objective function was weighted by the inverse probability weights 1/$\hat{P}_{i2}$. The intuition behind these weights was that respondents with characteristics similar to those of individuals missing due to attrition are up-weighted in the analysis and vice versa. The method of inverse-probability-of-attrition weighting corrects for selection bias under the assumption that conditional on observables in the first wave, second-wave participation is independent of health-related productivity and job stressors and resources in the second wave [67].

### Cost calculation

We estimated the costs of job stress based on the representative wave 1 dataset. Using the results of Eq. (1), we proceeded in four steps: *first*, job stress was defined as a binary variable taking the value 1 if job stressors exceeded job resources, which was the case if the net effect of workplace conditions on productivity losses was positive, and 0 otherwise (${\text{job}}\;{\text{stress}}_{i} = 1[ \beta_{1} \dot{R}_{i}^{j} + \beta_{2} \dot{S}_{i}^{j} > 0 ]$). *Second*, we converted the individual percentage productivity losses into monetary values by multiplying them with monthly earnings. This yielded the observed monthly production loss in Swiss francs (CHF) caused by health problems for an average employee in February 2014. The *third* step involved a counterfactual prediction. We predicted the health-related production loss that would have been observed if each employee experiencing job stress had a net workplace condition effect of zero, i.e., would not have been exposed to job stress. This yielded the predicted monthly production loss caused by health problems for an average employee in the absence of job stress. The *fourth* and final step consisted of taking the difference between the observed and the predicted production losses, which yielded the part of the health-related production loss attributable to job stress.

## Results

### Descriptive statistics of wave 1

Of the 3381 wave 1 participants, 54% were female, and the average age was 42.3 years (Table 1). Almost two-thirds were employed full-time (64%), and approximately one-fifth performed shift work (20%). In terms of job category, approximately 18% were self-employed, were firm owners or worked in independent professions, 31% were executive employees, 37% were non-executive employees, 17% were skilled workers, and 2.3% were unskilled manual workers.

**Table 1:** Descriptive statistics of the cross-sectional data (wave 1)

	Mean	SD
Health-related productivity losses (in %)	0.143	0.237
…due to absenteeism (in %)	0.034	0.14
…due to presenteeism (in %)	0.109	0.178
Job resources	3.85	0.66
Task-related job resources	3.77	0.83
Social job resources	3.94	0.75
Job stressors	2.03	0.51
Task-related job stressors	2.46	0.54
Social job stressors	1.6	0.64
Occupational self-efficacy	3.69	0.86
Socio-economic characteristics		
Women	0.54	0.50
Age	42.25	11.88
Single	0.37	0.48
Divorced	0.12	0.33
Number of children	0.49	0.86
Swiss native	0.84	0.37
Secondary education	0.56	0.50
Tertiary education	0.33	0.47
Job categories		
Firm owner, independent profession	0.11	0.32
Self-employed tradesperson	0.07	0.25
Executive employee	0.32	0.46
Non-executive employee	0.37	0.48
Skilled manual worker	0.17	0.38
Unskilled worker	0.02	0.15
Job characteristics		
Working full-time	0.64	0.48
Shift work	0.20	0.40
Managerial function	0.40	0.49
Tenure (in months)	115	110
Office size (number of persons)	3.29	2.52
Monthly income (CHF)	6149	2960
Industry		
Agriculture, forestry, fishing	0.03	0.18
Manufacturing	0.15	0.35
Construction	0.07	0.25
Trade and repair services	0.14	0.35
Transportation and storage	0.04	0.20
Accommodation and food service industry	0.04	0.20
Information and communication	0.03	0.18
Financial and insurance industry	0.06	0.24
Real estate, administrative and support services	0.04	0.20
Science and research	0.08	0.27
Public administration and defense	0.05	0.22
Education	0.07	0.26
Health, social work services	0.14	0.34
Arts	0.06	0.24
Home–work interference (std.)	0.37	0.40
Chronic conditions^a^		

^a^Because of space constraints, the sample characteristics on chronic health conditions are shown in Table A.2 in the Appendix

**Fig. 1:**
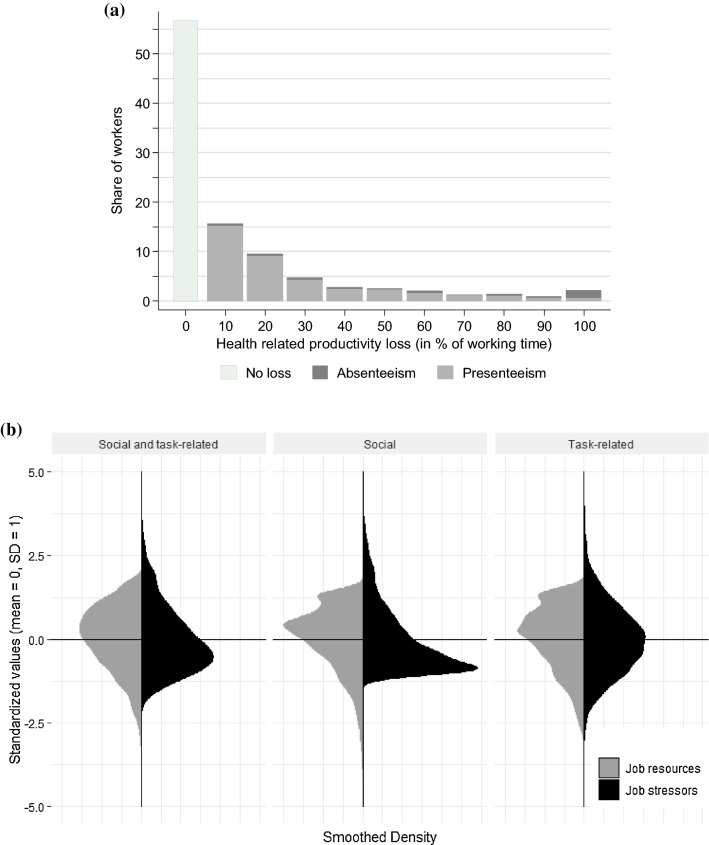
Distribution of key variables

The average health-related productivity losses amounted to 14.3% of the working time, corresponding to 6 h per week for a full-time employee. At 10.9%, presenteeism had a more important role than absenteeism (3.4%). These findings are in line with those of other studies using the WPAI (e.g., [68]). Moreover, Fig. 1a shows that 65% of the participants reported health-related productivity losses of zero. Of those with a non-zero loss, the majority reported a loss between 10 and 20%, corresponding to 4–8 h per week. On average, job resources (*M* = 3.85, SD = 0.66) were higher than job stressors (*M* = 2.03, SD = 0.51), and this difference was more pronounced for social than for task-related job stressors and resources. Furthermore, both job stressors and job resources exhibited a distinctive asymmetrical distribution with opposite skewness, with the majority of employees reporting above-average resources and below-average stressors (Fig. 1b).

### Correcting for selective attrition in wave 2

Table 2 reports the scale of selective attrition in the wave 1–2 subsample and shows how well the inverse-probability-of-attrition weights performed in adjusting for it. Comparing the characteristics between the wave 1 sample (column 1) and the unweighted wave 1–2 subsample (column 2) suggests that attrition was non-random as participation in the second wave was significantly related to age, office size, working in the art sector, and absenteeism. In particular, participants in the second wave were on average younger (43 vs. 42.3 years), worked in smaller offices (9.7 vs. 3.3 co-workers), showed higher absenteeism (2.5% vs. 3.4%) and were more likely to work in the art sector (2.6% vs. 6%) than participants in the first wave.

**Table 2:** Attrition and the results of inverse-probability-of-attrition weighting

	Mean values 2014 (time of wave 1)		
	Wave 1 participants	Wave 1–2 participants	
		Unweighted	Weighted (ipw)
Health-related productivity losses	0.143	0.127	0.138
…due to absenteeism	0.034	0.025**	0.031
…due to presenteeism	0.109	0.102	0.107
Job stressors	2.026	2.021	2.022
Social job stressors	1.595	1.602	1.588
Task-related job stressors	2.456	2.439	2.456
Job resources	3.851	3.874	3.854
Social job resources	3.936	3.945	3.934
Task-relates job resources	3.765	3.802	3.774
Occupational self-efficacy	3.687	3.703	3.691
Age	42.26	43.06*	42.13
Office size (number of persons)	3.287	9.666***	8.708***
Industry sector: arts	0.06	0.026***	0.039**
*N*	3381	1515	1515

This table compares the characteristics at wave 1 (2014) between the overall wave 1 sample (column 1) and the wave 1–2 subsample, once without weighting (column 2) and once with inverse-probability-of-attrition weighting (column 3). The upper panel presents the averages of our key variables. The lower panel presents the average values of covariates in which wave 1 and wave 1–2 participants show significant differences

***p < 0.001, **p < 0.05, *p < 0.01

The comparison of the characteristics between the wave 1 sample (column 1) and the weighted wave 1–2 subsample (column 3) illustrates that inverse-probability-of-attrition weighting is capable of reducing the differences between the two samples considerably. Small differences only remain with respect to office size and the probability of working in the art sector.

### Productivity effect of job stressors and job resources

Table 3 presents the main regression results using cross-sectional wave 1 data, with the first five models building up from simple correlations (CS-1) to the fully specified model presented in column five (CS-5). Column six (FE-5) shows the results of the fully specified fixed effects model based on longitudinal data (waves 1–2). Finally, columns seven and eight (CS-6 and FE-6) present the effects of task-related and social job stressors and resources on productivity separately.

**Table 3:** Effects of job stressors and resources on health-related productivity losses

Dependent variable	Health-related productivity losses (in % of working time)								
	CS-1	CS-2	CS-3	CS-4	CS-5	FE-5		CS-6	FE-6
Job resources ($\dot{R}^{j}$)	− 1.716*** (0.597)	− 1.528** (0.647)	− 1.558** (0.619)	− 1.546** (0.616)	− 1.310** (0.625)	− 1.208 (1.282)	$\dot{R}^{{j,\;{\text{social}}}}$	− 0.997* (0.572)	− 1.021 (1.096)
Job stressors ($\dot{S}^{j}$)	4.442*** (0.651)	4.864*** (0.655)	4.071*** (0.675)	4.049*** (0.673)	4.009*** (0.674)	3.812*** (1.222)	$\dot{R}^{{j, \;{\text{task}}}}$	− 0.405 (0.408)	− 0.378 (1.389)
							$\dot{S}^{{j,\;{\text{social}}}}$	1.929*** (0.412)	2.621** (1.157)
							$\dot{S}^{{j,\;{\text{task}}}}$	1.346** (0.561)	2.155** (1.071)
Region FEs (canton)	–	Yes	Yes	Yes	Yes	Yes		Yes	Yes
Socio-econ. and job char.	–	Yes	Yes	Yes	Yes	Yes		Yes	Yes
Private stressors	–	–	Yes	Yes	Yes	Yes		Yes	Yes
Chronic diseases	–	–	–	Yes	Yes	Yes		Yes	Yes
Self-efficacy					− 0.927** (0.435)			− 0.864*** (0.334)	
Year and individual FEs	–	–	–	–	–	Yes		–	Yes
Mean (SD)	14 (24)	14 (24)	14 (24)	14 (24)	14 (24)	14 (24)		14 (24)	14 (24)
Number of observations	3381	3381	3381	3381	3381	3026		3381	3026
Number of regressors	2	79	82	90	91	72		93	74
Adjusted *R*^2^	0.054	0.082	0.132	0.137	0.138	–		0.143	–
*p* value (*F* statistic)	< 0.001	< 0.001	< 0.001	< 0.001	< 0.001	< 0.001		< 0.001	< 0.001
*ρ*	–	–	–	–	–	0.767		–	0.766
Estimation method	OLS	OLS	OLS	OLS	OLS	FE		OLS	FE
Data	Wave 1	Wave 1	Wave 1	Wave 1	Wave 1	Wave 1–2		Wave 1	Wave 1–2
Elasticity $\epsilon_{\text{r}}$	− 0.695	− 0.618	− 0.631	− 0.626	− 0.53	− 0.527	$\epsilon_{\text{r,social}}$	− 0.472	− 0.418
Elasticity $\epsilon_{\text{s}}$	1.211	1.326	1.11	1.104	1.093	1.099	$\epsilon_{\text{r,task}}$	− 0.169	− 0.155
							$\epsilon_{\text{s,social}}$	0.302	0.483
							$\epsilon_{\text{s,task}}$	0.799	0.721

Robust standard errors are given in parentheses. ***p < 0.001, **p < 0.05, *p < 0.01. $\dot{R}^{j}$ and $\dot{S}^{j}$ are the standardized values of job resources and stressors. $\dot{S}^{{j,\;{\text{task}}}} , \dot{S}^{{j, \;{\text{social}}}} ,\dot{R}^{{j,\;{\text{task}}}}$ and $\dot{R}^{{j,\;{\text{social}}}}$ are the standardized values of task-related stressors, social stressors, task-related resources and social resources. Columns CS-1 to CS-6 show OLS estimates based on the wave 1 cross-sectional data. Columns FE-5 and FE-6 show fixed effects (FE) estimates based on the longitudinal wave 1–2 panel data while applying inverse-probability-of-attrition weighting. $\epsilon_{\text{s}}$ and $\epsilon_{\text{r}}$ denote the elasticity of health-related productivity losses with respect to job stressors and resources. The *p* values for the hypothesis: $\dot{S}^{{j,\;{\text{task}}}} = \dot{S}^{{j,\;{\text{social}}}}$ and $\dot{R}^{{j,\;{\text{task}}}} = R^{{j,\;{\text{social}}}}$ are 0.586 and 0.362 for CS-6, and 0.780 and 0.327 for FE-6. The statistical power to detect the effect of $\dot{R}^{j}$ (in FE-5), $\dot{R}^{{j, \;{\text{social}}}}$ and $\dot{R}^{{j,\;{\text{task}}}}$ (in FE-6) is only 46%, 45% and 23%, respectively

The results for the cross-sectional data show that (CS-1) a one-standard-deviation increase in job stressors is associated with an increase in health-related productivity losses of 4.4 percentage points (95% CI 3.2–5.7), and a one-standard-deviation increase in job resources is associated with a decrease in health-related productivity losses of 1.7 percentage points (95% CI − 0.5 to − 2.9). Adding socio-economic and job characteristics (CS-2) increases the point estimate of job stressors to 4.8 (95% CI 3.6–6.1) but decreases the point estimate of job resources to − 1.5 (95% CI − 0.3 to − 2.8). As expected, including family conflicts (CS-3) drives down the point estimate of job stressors to 4.1 (95% CI 2.7–5.4), while the point estimate of job resources remains unchanged. Adjusting for chronic diseases (CS-4) leaves both coefficients nearly unchanged, implying that chronic conditions are related neither to job stressors nor to resources. Finally, adding occupational self-efficacy (CS-5) reduces the point estimate of job resources to − 1.3 (95% CI − 0.1 to − 2.5) and, to a lesser extent, the point estimate of job stressors to 4 (95% CI 2.7–5.3). This implies a positive correlation between occupational self-efficacy and both job stressors and resources. One explanation might be that individuals with a high level of occupational self-efficacy may also seek job tasks (or jobs) that are more challenging and demanding, as they feel capable of mastering them, and such jobs are typically combined with high job resources (e.g., [69]). Also note that occupational self-efficacy significantly reduces productivity losses due to absenteeism and presenteeism.

The point estimates of the fixed effects regression based on wave 1–2 (FE-5) appear statistically equivalent to those of the fully specified model using wave 1 (CS-5). A one-standard-deviation increase in job stressors leads to an increase in productivity losses of 3.8 percentage points (95% CI 1.4–6.2), whereas a one-standard-deviation increase in job resources leads to a decrease in productivity losses of − 1.2 percentage points (95% CI 1.3 to − 3.7). While the point estimates are statistically identical, the standard errors have nearly doubled due to the smaller sample size, which renders the coefficient of job resources insignificant.

Converted into elasticities, our results suggest an elasticity of health-related productivity losses of 1.09 with respect to job stressors and an elasticity of − 0.53 with respect to job resources (as shown in the last two rows).

The results of distinguishing between social and task-related job stressors and job resources are presented in the last two columns of Table 3 with CS-6, which present OLS and FE-6 fixed effects regression results. The coefficients on both task-related and social job stressors are positive and statistically significant in both models. Moreover, although productivity losses seem to be slightly more affected by task-related than by social job stressors, the hypothesis of equal effects cannot be rejected (see Table 3, notes). A similar pattern emerges for job resources, as we cannot reject the hypothesis that social and task-related job resources affect productivity equally. However, in the fixed effects model (Table 3, FE-6), neither social nor task-related resources are statistically significant. The elasticity of lost productivity with respect to social job stressors ranged between 0.30 and 0.48, and that with respect to task-related stressors ranged between 0.72 and 0.8.

In terms of the comparability between the wave 1 and wave 1–2 results (OLS vs. fixed effects regression), it should be kept in mind that although the weights reduced sample differences considerably (see Table 2), we cannot rule out the possibility that a certain attrition bias is still present. Nonetheless, the fact that both models yield statistically equivalent results can be interpreted as strong evidence that omitted time-invariant variables in the cross-sectional data hardly bias the results.

### Robustness checks

We performed a set of robustness checks. The first robustness check was related to the fact that the dependent variable is strictly non-negative and contains a large mass of zeros, which might lead to biased estimates when estimated by OLS. We therefore transformed the dependent variable into a count variable corresponding to the weekly number of hours lost due to health problems, and we tested the robustness of the baseline results with respect to the use of a negative binomial model (NBM), which is especially suited to account for zero-inflated over-dispersed count data [70]. The results are shown in the first two columns of Table 4. Column 1 (C1) replicates our baseline estimates (Table 3, CS-5) using the transformed dependent variable. C2 presents the NBM results based on the same covariate specification. The comparison shows that the models lead to very similar marginal effects. The OLS point estimates lie between the marginal effects estimated by the NBM at mean characteristics and the average marginal effects. This is strong evidence that our results are not driven by the choice of the model. The next robustness check refers to the specification of both the covariates and the main explanatory variables. The specification in C3 differs from the baseline model, as it allows for interaction effects between gender, age, education and industry sector and includes the gender-age-education-sector distribution with sixteen sectors and five education categories. Again, the resulting point estimates are virtually identical to our baseline estimates. The last two robustness checks refer to the functional form of the relationship between the dependent variable and job stressors and resources. Model C4 estimates different effects for below- and above-average job resources and stressors, and C5 tests for a quadratic form of the relationships providing no evidence for a significant non-linear relationship.

**Table 4:** Robustness checks

Dependent variable	Health-rel. prod. loss (h) (count variable)		Health-related productivity losses (%) (continuous variable)			
	C1	C2		C3	C4	C5
Point estimates			Point estimates			
Job resources ($\dot{R}^{j}$)	− 0.453** (0.260)	− 0.099** (0.047)	Job resources ($\dot{R}^{j}$)	− 1.228** (0.626)	− 1.848*** (0.517)	− 0.734 (1.198)
Job stressor ($\dot{S}^{j}$)	1.536*** (0.281)	0.306*** (0.042)	Job stressor ($\dot{S}^{j}$)	4.027*** (0.671)	3.083*** (0.540)	2.989** (1.332)
Marginal effects						
${\text{mfx}}_{{R^{j} }}$ at means		− 0.371*** (0.177)	$\dot{R}^{{j^{2} }}$		− 0.267 (0.345)	
${\text{mfx}}_{{S^{j} }}$ at means		1.142*** (0.154)	$\dot{S}^{{j^{2} }}$		0.476 (0.387)	
Mean ${\text{mfx}}_{{R^{j} }}$		− 0.576** (0.282)	$\dot{R}^{j} \cdot I(\dot{R}^{j} > 0)$			− 0.677 (1.630)
Mean ${\text{mfx}}_{{S^{j} }}$		1.774*** (0.270)	$I(\dot{R}^{j} > 0)$			− 0.772 (1.508)
			$\dot{S}^{j} \cdot I(\dot{S}^{j} > 0)$			2.830 (1.813)
			$I(S^{j} > 0)$			− 1.898 (1.604)
Mean	6	6		14	14	14
SD	10	10		24	24	24
Nr observations	3381	3381		3381	3381	3381
Nr regressors	91	91		101	93	95
Adjusted *R*^2^	0.137	0.027		0.097	0.092	0.097
*p* value (*F* stat.)	< 0.001	< 0.001		< 0.001	< 0.001	< 0.001
Baseline controls	Yes	Yes		–	Yes	Yes
Alternative controls	–	–		Yes	–	–
Model	OLS	NBM		OLS	OLS	OLS

Robust standard errors are given in parentheses. ***p < 0.001, **p < 0.05, *p < 0.01. $\dot{R}^{j}$ and $\dot{S}^{j}$ denote the standardized values of job resources and job stressors. $I(\dot{R}^{j} > 0)$ and $I(\dot{S}^{j} > 0)$ are dummy variables indicating resources and stressors that are above the mean

### Interaction effects

In our baseline model (Eq. 1), we assumed that job stressors affect health-related productivity losses independently of the level of job resources and vice versa and that both the impact of job stressors and the impact of job resources do not depend on the level of occupational self-efficacy. We relaxed these assumptions and estimated two additional models. In Model 1 (CS-7), we added an interaction term between job stressors and resources to the baseline model (CS-5). In Model 2 (CS-8), we additionally included interaction terms between job stressors, job resources, and occupational self-efficacy (Table 5).

**Table 5:** Heterogeneous effects

Dependent variable:	Health-related productivity losses (in %)	
	CS-7	CS-8
Job resources ($\dot{R}^{j}$)	− 1.197* (0.622)	− 1.148* (0.624)
Job stressor ($\dot{S}^{j}$)	3.704*** (0.679)	3.662*** (0.690)
Personal resources ($\dot{R}^{p}$)	− 0.979** (0.438)	− 0.427 (0.471)
$\dot{R}^{j} \times \dot{S}^{j}$	− 0.704 (0.432)	− 0.393 (0.422)
$\dot{R}^{j} \times \dot{R}^{p}$		0.247 (0.479)
$\dot{S}^{j} \times \dot{R}^{p}$		− 0.302 (0.685)
$\dot{R}^{j} \times \dot{S}^{j} \times \dot{R}^{p}$		0.657** (0.309)
Baseline controls	Yes	Yes
Mean	14	14
Standard deviation	24	24
Number of observations	3381	3381
Number of regressors	92	95
Adjusted *R*^2^	0.139	0.141
*p* value (*F* statistic)	0	0

**Fig. 2:**
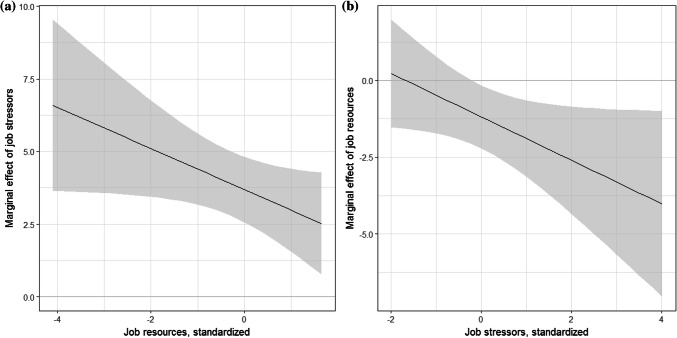
Marginal effects of job stressors and resources allowing for interaction effects. Notes: **a** The marginal effects of job stressors on lost productivity depending on job resources. **b** The marginal effects of job resources on lost productivity depending on job stressors. The estimates and the 90% CI are based on the results shown in column 1 of Table 5

**Fig. 3:**
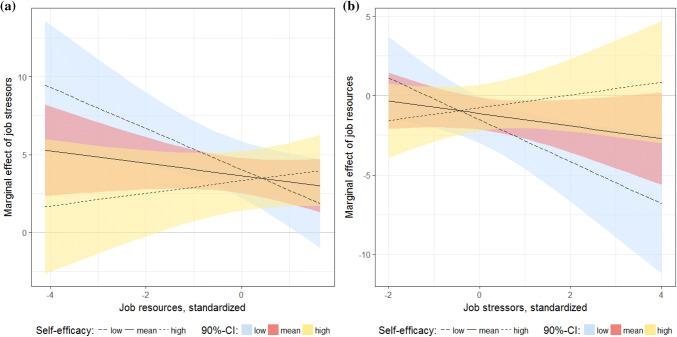
Marginal effects of job stressors and job resources depending on occupational self-efficacy. **a** The marginal effects of job stressors on health-related productivity losses depending on job resources and at low (1st decile), average and high (9th decile) values of occupational self-efficacy. **b** The marginal effects of job resources on health-related productivity losses depending on job stressors at low (1st decile), average and high (9th decile) values of occupational self-efficacy. The estimates and the 90% CI are based on the results shown in column 2 of Table 5

Robust standard errors are given in parentheses. ***p < 0.001, **p < 0.05, *p < 0.01. $\dot{R}^{j}$ and $\dot{S}^{j}$ denote the standardized values of job resources and job stressors. $\dot{R}^{p}$ denotes the standardized value of occupational self-efficacy. Graphic representations of the results in C1 and C2 are shown in Figs. 2 and 3

Comparing CS-7 (Table 5; Fig. 2) with our baseline results in CS-5 (Table 3) shows that the impact of job resources on productivity—when estimated at average stress levels—is similar, although somewhat smaller than the constant resource effect estimated by the baseline model. The same applies to the effect of job stressors. However, the coefficient of the interaction term turns out negative (and only closely misses the 10% significance level), indicating a decreasing marginal effect of job stressors on health-related productivity losses with increasing levels of job resources. Furthermore, the productivity effect of job stressors is significant at all levels of job resources and approximately twice as large at the minimum level than at the maximum level of job resources (Fig. 2a). By contrast, job resources affect work productivity only at higher levels of job stressors (> fourth decile). In summary, the productivity effect of a change in job stressors is larger for individuals with low, rather than high, job resources. In contrast, the effect of a change in job resources is larger for individuals with high compared to low stressors.

The results of Model 2 (CS-8) are presented in Table 5 and, for easier interpretation, in Fig. 3. Graph (a) shows the marginal effects of job stressors on lost productivity depending on job resources at low (first decile), medium (mean) and high (ninth decile) levels of occupational self-efficacy. Graph (a) shows that at low levels of occupational self-efficacy, the marginal effects of job stressors heavily depend on the level of job resources: the lower the job resources, the larger the negative productivity effect of job stressors. With increasing levels of occupational self-efficacy, however, this relationship becomes weaker until it disappears at about the sixth decile of occupational self-efficacy. Graph (b) shows the marginal effects of job resources. In contrast to job stressors, job resources do not affect every individual’s productivity loss. Positive effects of job resources are found for individuals who have above-average job stressors and below-average occupational self-efficacy. The effects for individuals with low occupational self-efficacy who face high job stressors are largest. In sum, individuals with low occupational self-efficacy are the most vulnerable in the sense that negative and positive changes in job stressors and resources have the biggest impact on work productivity in the expected direction.

### Costs of work stress

Table 6 presents our estimates on the costs of job stress due to absenteeism and presenteeism. Our results suggest that job stress accounts for 23.8% of the total health-related production losses, which, in monetary terms, corresponds to CHF 195 per person and month. This corresponds to 3.2% of the average monthly earnings in Switzerland.

**Table 6:** Average monthly per capita costs of job stress

Average monthly health-related production loss	CHF	% of observed
Observed	820	
Predicted (scenario: no job stress exists)	625	76.2
(standard error)	(240)	
Attributable to job stress	195	23.8

Job stress is positive if the net effect of job stressors and job resources on productivity losses is positive. This applies to 64.5% of the employees in our data set. Production losses correspond to productivity losses (in % of working time) multiplied by monthly earnings

## Discussion

We estimated the impact of job stressors and job resources on productivity losses due to sickness absenteeism and presenteeism based on a representative survey of Swiss employees conducted in 2014 and 2015. First, we found that health-related productivity losses increase with an increase in job stressors and decrease with an increase in job resources, with social and task-related stressors and resources being equally important determinants. Second, the analysis of heterogeneous effects revealed that an increase in job stressors is especially harmful if job resources are low. These effects are even more pronounced if occupational self-efficacy is low as well. On the other hand, an increase in job resources is most effective in reducing health-related productivity losses if job stressors are high and occupational self-efficacy is low. Third, the results of a counterfactual analysis suggest that job stress (defined as job stressors exceeding job resources) accounts for 23% of the total health-related productivity losses due to absenteeism and presenteeism. This corresponds to CHF 195 per person and month.

Our findings contribute to studies on the effects of positive and negative social aspects of work on presenteeism and absenteeism. In line with research showing that social aspects of work may be especially relevant to employee health and organizational behavior [25, 46], we found that social stressors and resources at work are important determinants of health-related productivity losses due to absenteeism and presenteeism in addition to task-related job stressors and resources. Moreover, we found that social and task-related stressors have direct and equal effects on health-related productivity losses, and while social resources remain a significant predictor, task-related resources do not. If employees work under unfavorable work conditions characterized by high levels of job demands, do not feel appreciated or respectfully treated at work, or lack social support, health-related productivity losses due to absenteeism and presenteeism might increase. This behavior can be seen as a method of employees restoring equity in the employee-organization relationship, as proposed by the social exchange perspective [18]. These results have scientific and practical implications. Our findings suggest that both social and task-related factors should be considered in future studies and in planning interventions aiming to reduce health-related productivity losses by improving workplace conditions.

As expected, our results confirm that job resources buffer the negative effects of job stressors on productivity losses. These findings are in line with the buffering hypothesis of the JDC model as well as with the postulation that high-strain jobs, characterized by a combination of high job demands and low resources, should see the most harmful effects, while the combination of high demands and high resources is considered to be the most beneficial (active job) [8]. Moreover, our results show that an increase of 1% in job stressors results in a larger effect on health-related productivity losses than a decrease of 1% in job resources. These results are in line with those of previous studies showing that negative conditions and events typically have stronger effects than good conditions [71]. This implies that an increase in demands at work should always be accompanied by an even larger increase in job resources in order to prevent the negative consequences regarding health-related productivity impairments.

Our results also show that not only job resources but also occupational self-efficacy buffer the negative effects of job stressors on health-related productivity losses. Furthermore, we find that employees with a simultaneous lack of personal and job resources are the most vulnerable with respect to an increase in job stressors. This finding is in line with the vicious cycle postulated by the COR model: individuals who lack resources are more vulnerable to resource loss and less capable of resource gain. We also find that employees with low personal resources facing high job stressors are the ones who would profit the most from an increase in job resources. This is in line with the “gain paradox principle” of the COR model [35], stating that resources are even more important when resource losses are high.

We do not find a significant productivity effect of an increase in job resources for employees with high personal resources and low level of job stressors. Therefore, an increase in job resources without a reduction in job stressors may not always be sufficient to reduce health-related productivity losses.

We add to the economic literature by estimating the total health-related productivity loss due to unfavorable job conditions. Our estimated productivity loss of CHF 195 per person and month may seem modest at first. However, extrapolation indicates that job stress may have cost Swiss companies up to CHF 10 billion in 2014, corresponding to 1.7% of the gross domestic product. This emphasizes the economic importance of interventions aiming to improve work conditions in general and the balance between work demand and resources.

Our study has several methodological and theoretical strengths. *First*, the cross-sectional data were representative of Swiss employees with respect to gender, age, region, and industry branch. *Second*, we tested the robustness of our cross-sectional results using longitudinal data, as this allowed the application of methodologically superior panel-data estimation methods. *Third*, we included several task-related and social work conditions and explored the relevance of positive and negative social aspects at work beyond the task-related aspect. *Fourth*, in addition to job resources, we considered personal resources—occupational self-efficacy—and explored interaction effects with job stressors and job resources.

Several limitations need to be taken into account. *First*, self-reported measures such as the WPAI-GH may suffer from social desirability and recall bias. While a recall bias is unlikely, given the 1-week recall period of WPAI-GH, a social desirability bias is likely to be present. Studies comparing self-reported with company-registered absenteeism show that employees tend to underreport absenteeism [22]. If this were due to social desirability, we would also expect employees to underreport presenteeism. While this would lead to an underestimate of the magnitude of health-related productivity losses, it would not necessarily bias the validity of the associations between workplace conditions and health-related productivity losses. A *second* shortcoming related to the WPAI-GH is that it has not (yet) been validated against objective work productivity data. We thus do not know whether an employee-reported productivity impairment of 10% translates into a 10% loss of an employee’s value to the employer. A study comparing self-reported measures from the Work Limitations Questionnaire (WLQ) with objective productivity outcomes found that a self-reported 10% health-related limitation at work translated into a 4–5% reduction in work output. However, the generalizability of these results is unclear because the study was carried out in a single work setting and did not consider quality of work [14]. If this overestimation in self-reporting of productivity losses applied to our data, it would imply an overestimation in our job stress-induced productivity losses. There is a clear need for more research on the extent to which employee-reported productivity measures translate into production losses for employers. A *third* limitation relates to the high dropout rate in the second wave of the survey. Although we show that the inverse-probability-of-attrition weights are capable of correcting for selective attrition to a large extent, we cannot rule out the possibility that our panel data estimations are still biased.

Our results suggest that improvements in work conditions could help organizations to reduce previously undetected productivity losses by implementing programs targeting an improved balance between job stressors and job resources. We also show that an increase in job demands affects employees to different degrees depending on their levels of job and personal resources and that not everyone benefits from increased job resources. This finding highlights the need for organizations to take a tailored approach by providing additional attention to the most vulnerable employees. Moreover, our data suggest that job stressors and resources as well as health-related productivity losses vary greatly across occupations. Our sample size prevents the estimation of occupation specific effects though, offering an opportunity for future research.

## Electronic supplementary material

Below is the link to the electronic supplementary material.

Supplementary material 1 (DOCX 23 kb)
