# Supplementary material for: Who gains the most from improving working conditions? Health-related absenteeism and presenteeism due to stress at work
Source: Eur J Health Econ. 2019 Jul 15;20(8):1165–80. doi: 10.1007/s10198-019-01084-9 (PMC6803571; doi:10.1007/s10198-019-01084-9)
Supplement: Supplementary file 1 — Supplementary material 1 (DOCX 23 kb) [file 10198_2019_1084_MOESM1_ESM.docx]

## Appendix

## . Additional tables

Table A.1: Cronbach's alpha

| ***Panel A:*** *Work-related stressors:* | |  |
| --- | --- | --- |
|  | Time pressure | 0.83 |
|  | Uncertainty | 0.68 |
|  | Performance constraints | 0.71 |
|  | Qualitative overload | 0.81 |
|  | Social stressors supervisor | 0.86 |
|  | Social stressors colleagues | 0.84 |
| Social job stressor ($\dot{S}^{j,soc}$) | | 0.88 |
| Task-related job stressor ($\dot{S}^{j,task}$) | | 0.81 |
| Overall job stressor ($\dot{S}^{j}$) | | 0.88 |
| ***Panel B:*** *Work-related resources:* | |  |
|  | Job control | 0.87 |
|  | Task significance (single item) |  |
|  | Support from supervisor | 0.88 |
|  | Appreciation (single item) |  |
| Social job resource ($\dot{R}^{j,soc}$) | | 0.88 |
| Task-related job resource ($\dot{R}^{j,task}$) | | 0.87 |
| Overall job resource ($R^{j}$) | | 0.86 |
| ***Panel C****: Personal resources and private stressors:* | | |
|  | Occupational self-efficacy | 0.88 |
|  | Home-work interference | 0.75 |

Table A. 2: Wave 1 sample characteristics, continued

| *Chronic conditions:* | | Mean share | SD |
| --- | --- | --- | --- |
|  | Asthma | 0.013 | (0.11) |
|  | Diabetes | 0.019 | (0.14) |
|  | Osteoarthritis, rheumatoid arthritis | 0.026 | (0.16) |
|  | Osteoporosis | 0.007 | (0.08) |
|  | Chronic bronchitis or emphysema | 0.008 | (0.09) |
|  | Kidney disease | 0.008 | (0.09) |
|  | Cancer or tumor | 0.007 | (0.09) |
|  | Allergies | 0.03 | (0.17) |
|  | Permanent injury after accident | 0.023 | (0.15) |

## Estimating inverse-probability-of-attrition weights

The inverse-probability-of-attrition weights are estimated based on a logit model, with the dependent variable being a binary variable indicating second-wave participation. This indicator variable is regressed on a series of observed individual and job characteristics in time period 1 (year 2014). Subsequently, probabilities $\hat{P}_{i2}$are fitted, transformed to the inverse 1/$\hat{P}_{i2},$ and then used to weight the objective function (in our case, the fixed effects model). The covariate selection was performed based on the Akaike information criterion (AIC) and the Bayesian information criterion (BIC). The model is specified as follows:

$P\left( D_{i2}=1 \right)=\alpha+\beta_{0}\mathbf{X'}_{i1}+\beta_{1}\mathbf{J'}_{i1}+\beta_{2}\mathbf{H'}_{i1}^{ph}+\beta_{3}\mathbf{H'}_{i1}^{m}+\beta_{4}\mathbf{RS}_{i1}+\theta_{r}+\epsilon_{i1}$ (A.1)

with $D_{i2}$ denoting second-wave participation of individual $i$. The vector $\mathbf{X'}$ includes demographic and socio-economic characteristics such as gender, age, age squared, Swiss citizenship, marital status (3 dummy variables), number of children and highest educational attainment (6 dummy variables). The vector $\mathbf{J'}$ includes a number of labor market and job characteristics. It contains 7 occupation dummies, 16 industry dummies, office size and office size squared, years of service, dummy variables indicating 10 wage categories, the average number of working hours during the past three months and dummy variables for part-time employment, shift work and managerial function. The vector $\mathbf{H'}^{ph}$ captures physical health. It contains a set of 16 dummy variables indicating current medical treatment for psychosomatic and non-psychosomatic chronic diseases and 16 dummy variables indicating the presence of these chronic diseases during the past year. It also includes self-rated overall health. Mental health and well-being are captured by the vector $\mathbf{H'}^{m}$, which includes job satisfaction, work-home interference, problems with having a clear head after work, general nervousness, grumpiness, getting angry easily, reacting irritably and having weak nerves. It also includes three variables indicating the level of sleeping disorders with respect to having problems getting to sleep, staying asleep and waking up early. Finally, $\mathbf{RS}$ includes all possible interaction terms between task-related job stressors, social job stressors, task-related job resources, social job resources, private stressors and private resources. It also includes the second and third polynomial terms of all stressors and resources. Finally, cantonal fixed effects are denoted by $\theta_{r}$.
